# Ramsey hunt syndrome after antimonial treatment for American Cutaneous Leishmaniasis

**DOI:** 10.1590/0037-8682-0012-2020

**Published:** 2020-11-13

**Authors:** Melissa de Sousa Melo Cavalcante, Karina López Rodríguez, José Alejandro Lazo Diéguez, Luciana Mendes dos Santos, Maria das Graças Vale Barbosa Guerra, Jorge Augusto de Oliveira Guerra

**Affiliations:** 1 Fundação de Medicina Tropical Doutor Heitor Vieira Dourado, Manaus, AM, Brasil.; 2 Universidade do Estado do Amazonas, Programa de Pós-Graduação em Medicina Tropical, Manaus, AM, Brasil.; 3 Universidade Federal do Amazonas, Faculdade de Medicina, Manaus, AM, Brasil.

**Keywords:** Leishmaniasis, Ramsay Hunt Syndrome, Herpes zoster, Facial paralysis

## Abstract

Ramsay Hunt Syndrome (RHS), also known as herpes zoster oticus, is caused by the reactivation of varicella zoster virus (VZV) in the geniculate ganglion of the facial nerve. Herein, we report a case of Ramsey Hunt Syndrome in a patient after antimonial treatment for Cutaneous Leishmaniasis. The patient presented with microvesicles grouped on an erythematous base, starting in the neck and ascending towards the scalp margin on the right side of the head. The patient also developed grade V peripheral facial palsy the day after initiating the herpes zoster treatment, this outcome corroborated the assumption of Ramsey Hunt Syndrome.

## INTRODUCTION

Ramsay Hunt Syndrome (RHS) was first described by James Ramsay Hunt in 1907. The syndrome is characterized by a vesicular rash on the pinna, ipsilateral otalgia, and peripheral facial palsy on the same side of the head^1^. However, it can also presents signs and symptoms such as hypoacusis, nausea, vomiting, vertigo, and nystagmus related to proximity-related eighth cranial nerve affection^2^. RHS is the second most common cause of facial paralysis, after Bell’s palsy^3^. The incidence of RHS is approximately 5 cases/100,000 individuals and it primarily affects patients in the 20-30 year age group^4^. RHS is also often observed in elderly immunosuppressed patients^5^. 

## CASE REPORT

A 55-year-old male patient who was born in Manaus metropolitan area, in the Brazilian state of Amazonas, with no comorbidities, sought medical help at the leishmaniasis service of the Doctor Heitor Vieira Dourado Foundation for Tropical Medicine (FMT-HVD) in Manaus. He had completed treatment with a pentavalent antimonial drug for cutaneous leishmaniasis (CL) 40 days prior. A history and physical examination detected an active lesion with satellite papule in the patient’s right upper limb. He received outpatient treatment with a second cycle of antimonial application for 30 days. 

**FIGURE 1:**
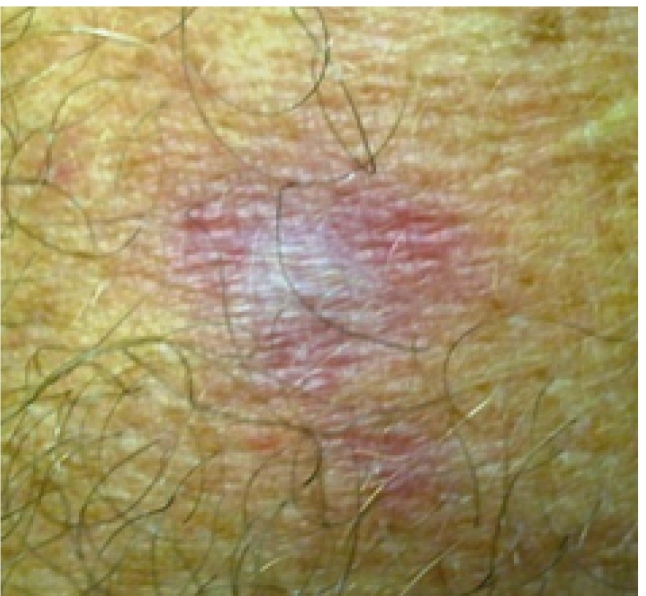
Lesion of cutaneous leishmaniasis in a initial stage of healing.

**FIGURE 2:**
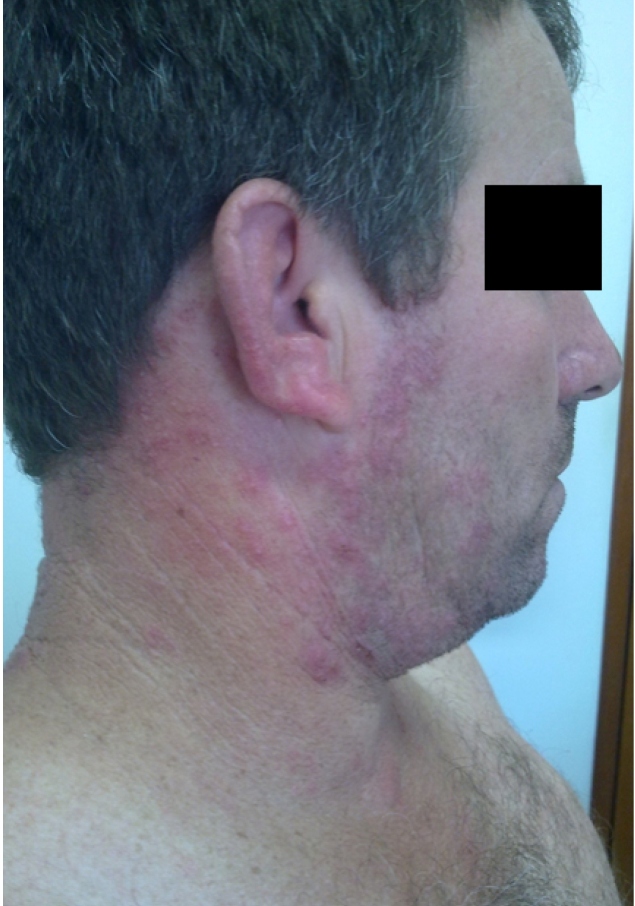
Microvesicles grouped on an erythematous-infiltrated base in the cervical region, right pinna and ear lobe, and scalp margin.

**FIGURE 3:**
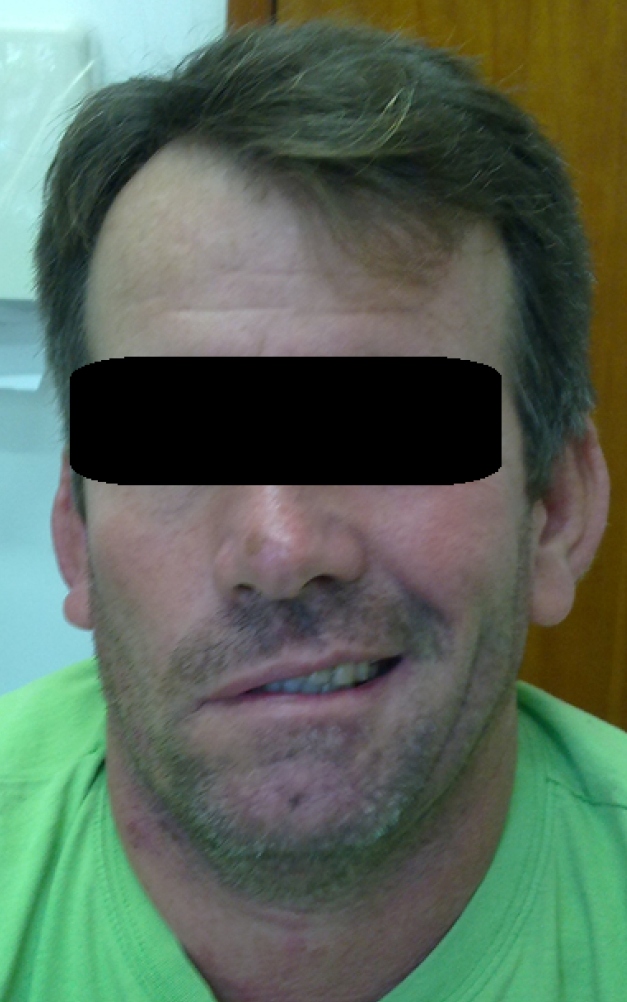
Grade V peripheral facial palsy after pentavalent antimonial treatment for cutaneous leishmaniasis.

Ten days after the end of treatment, the patient’s leishmaniasis lesion was healed (Figure 1). However, it presented with *microvesicles* *grouped* on an*erythematous base,* starting in the neck and ascended towards the scalp margin on the right side of the head. The *microvesicles* affected the patient’s pinna, external auditory canal, and right ear lobe, and were associated with severe torticollis-like pain in the nerve path (Figure 2). The patient received herpes zoster treatment with the following doses: 800 mg of acyclovir every 4 hours for 7 days, 25 mg of amitriptyline every 12 hours, and 500 mg + 30 mg of paco (paracetamol + codeine phosphate). He developed grade V peripheral facial palsy the day after initiating the herpes zoster treatment; this outcome corroborated the assumption of Ramsey Hunt Syndrome (Figure 3). Serology tests for human immunodeficiency virus (HIV) were negative. Corticosteroids (40 mg of prednisone/day), epitezan ointment, and hourly eye lubricant applications were added to the treatment. 

The patient reported improvement in the cervical pain at the end of the acyclovir-based treatment; however, he continued to have persistent headache and peripheral facial palsy symptoms. Following the gradual reduction of these symptoms, he was referred to another hospital in Manaus County for motor physical therapy treatment. The eye lubricant applications and analgesic medication were maintained, and the patient was referred to the neurology service for an outpatient follow-up where he presented with full neurological recovery. 

## DISCUSSION

RHS, is the second most common cause of non-traumatic facial palsy in adults^4^. In addition, it is often underdiagnosed, or misdiagnosed as Bell’s palsy, trigeminal neuralgia or otitis externa^4^. However, the evolution of RHS is more severe than that of Bell’s palsy and only 30% of RHS-affected patients achieve full recovery^3^. 

Clinical diagnosis of RHS is based on the signs and symptoms although VZV-specific serology can be indicated in atypical cases^1^. Although polymerase chain reaction (PCR) enables identification of the VZV genome^5^, these techniques cannot make a quick diagnosis early in the disease course. Tzanck’s cytodiagnosis is the most useful in emergency cases because of its fast application and simple interpretation. Additionally, while nuclear magnetic resonance imaging is a non-specific diagnostic technique, it is effective in assessing the prognosis of peripheral palsies^6^. 

RHS should be diagnosed as early as possible to enable the fast implementation of appropriate treatment and to avoid sequelae^5^. These complications include encephalitis, myelitis, and cranial and peripheral nerve palsies^2^. Early treatment with corticosteroids and antiviral drugs enables high rates of full facial nerve recovery within 6-12 months^7^. Patient characteristics, such as age, associated diseases, immunodeficiencies, and their clinical condition, can lead to a worse prognosis.

Herpes zoster association with antimonial treatment for leishmaniasis has also been reported with a possible secondary link to transient lymphopenia^8^. Hartzell et al.^9^ described a patient who developed aseptic meningitis and herpes zoster secondary to varicella zoster virus (VZV) during antimonial treatment for American Tegumentary Leishmaniasis.

Any individual who has had chickenpox is susceptible to RHS. The syndrome is not contagious; however, VZV reactivation can cause chickenpox in contacts who were not previously immunized against the virus. The infection in immunodeficient patients can be severe; therefore, recommendations include avoiding physical contact with individuals in the infection stage or those who have not been vaccinated, as well as with immunosuppressed individuals, newborns, and pregnant women^6^. 

This report presented the case of a previously healthy patient who developed a rare VZV-associated facial palsy after treatment with antimonial drugs for cutaneous leishmaniasis. The patient’s condition improved following the use of antiviral drugs and systemic corticosteroids. Early diagnosis and effective intervention resulted in the manifestation of his disease and the primary outcome was similar to the ones described in the literature. 
